# *Toxoplasma* effector GRA15-driven CCL5 secretion enhances brain parasite load through microvascular sequestration of phagocytes

**DOI:** 10.1128/mbio.03444-25

**Published:** 2026-01-13

**Authors:** Elena Afanaseva, Matias E. Rodriguez, Antonio Barragan

**Affiliations:** 1 Department of Molecular Biosciences, The Wenner-Gren Institute, Stockholm University 193207, Stockholm, Sweden; University of Geneva, Geneva, Switzerland

**Keywords:** (MeSH): host-pathogen, central nervous system protozoal infections, intracellular parasitism, blood-brain barrier, chemokines, leukocyte chemotaxis

## Abstract

*Toxoplasma gondii* exploits host phagocytes to disseminate systemically and establish infection in the central nervous system (CNS). Yet, the mechanisms governing the interaction between parasitized phagocytes and the brain endothelium remain elusive. Here, we show that *T. gondii* infection, but not parasite lysates, robustly induces transcriptional and secretory upregulation of the chemokine C–C motif ligand 5 (CCL5/RANTES) in primary brain endothelial cells and dendritic cells (DCs). This response was triggered by the parasite effector GRA15 through NF-κB signaling, while the effector TEEGR counteracted CCL5 induction in an MYR-translocon-dependent manner. In response to recombinant CCL5, infected DCs displayed increased hypermotility, chemotaxis toward CCL5 gradients, and enhanced transmigration across polarized endothelial monolayers. Intraperitoneally infected mice rapidly upregulated CCL5 in the blood and *Ccl5* expression in the cerebral microvasculature, thereby increasing the adhesion of parasitized DCs and cerebral parasite loads. Pretreatment of mice with recombinant CCL5 dramatically elevated the sequestration of infected DCs, while treatment with the selective chemokine receptor 5 (CCR5) antagonist Maraviroc reverted sequestration. Together, these findings reveal that *T. gondii* co-opts the host CCL5/CCR5 axis via GRA15-mediated signaling to promote leukocyte-dependent dissemination and early colonization of the CNS.

**IMPORTANCE:**

The intracellular parasite *Toxoplasma gondii* invades immune cells to spread through the circulatory system, eventually reaching the brains of humans and animals. It is not well understood how parasitized immune cells interact with blood vessel walls, a process that ultimately helps *Toxoplasma* colonize the brain tissue. We found that when Toxoplasma infects the cells lining the blood vessels (endothelium), these produce C-C motif chemokine ligand 5 (CCL5), a potent signaling and attractant molecule. CCL5 production was triggered by a parasite-derived secreted protein, GRA15. CCL5 activated and attracted infected immune cells. In mice, the levels of CCL5 increased quickly in the brain microvasculature after infection, helping the infected immune cells adhere to brain vessels. When the effect of CCL5 was pharmacologically blocked, fewer infected cells sequestered in the brain vessels, lowering the parasite loads. These findings reveal a mechanism through which *Toxoplasma* manipulates host cells to produce factors that facilitate its colonization of the brain.

## INTRODUCTION

The blood-brain barrier (BBB) is a selective, protective barrier that regulates the movement of substances between the bloodstream and the central nervous system (CNS). It is primarily formed by specialized endothelial cells that line the brain’s capillaries, tightly joined together by complex tight junctions (1). These endothelial cells restrict the passage of blood cells, microbes, toxins, and large molecules while allowing essential nutrients and gases to pass through. Thus, the BBB plays a critical role in maintaining the brain’s homeostasis and protecting it from harmful agents, such as microbial pathogens (2).

C-C motif chemokine ligand 5 (CCL5, also known as RANTES) is a chemokine involved in immune cell recruitment in multiple inflammatory conditions (3). It plays a key role in attracting immune cells such as T cells, monocytes, and dendritic cells (DCs) to sites of infection or injury. CCL5 binds to chemokine receptor 5 (CCR5) expressed on the surface of various immune cells, having a lower affinity for CCR1 and CCR3 (4). The CCL5/CCR5 interaction triggers signaling pathways that enhance immune cell migration, survival, and activation, including NF-kB, a pivotal mediator of inflammatory responses (3). Consequently, the CCL5/CCR5 axis is implicated in several diseases, such as HIV infection, where CCR5 serves as a co-receptor for viral entry, as well as in cancer, where hijacking of this axis can promote tumor progression and metastasis (5). Normally not expressed by the BBB endothelium, CCL5 can be expressed by endothelial cells upon inflammation (6). Rather, microglia and astrocytes have been identified as key producers of CCL5 in response to inflammation in the CNS (7). However, its specific functions in the brain remain enigmatic, with possible roles in the regulation of neuroendocrine processes, neurotransmission, and activation of immune cells in the parenchyma (8, 9).

In natural oral infections, *Toxoplasma gondii* first crosses the intestinal epithelium. Then, the fast-replicating tachyzoite stage spreads systemically to deeper tissues and traverses the BBB to reach the brain parenchyma, where it can ultimately cause the most severe pathology in humans, particularly in immunosuppressed individuals or developing fetuses. However, primary infection in healthy individuals, including colonization of the CNS, occurs in a remarkably stealthy manner, often asymptomatically or with mild symptoms. The infection then transitions into a chronic, asymptomatic phase marked by persistent parasite cysts in the CNS of humans and other intermediate hosts (10, 11).

One strategy that *T. gondii* employs to disseminate within vertebrate hosts is the hijacking of immune cells, such as DCs, monocytes, and macrophages, as vehicles for transport through the bloodstream and lymphatic system (12, 13). This mechanism enables *T. gondii* to evade immune detection and spread systemically, ultimately reaching the BBB. Once inside host cells, *T. gondii* elevates the migration of phagocytes by inducing a hypermigratory phenotype (14) that aids the parasite’s dissemination to peripheral organs, including the CNS (15, 16). Parasitized phagocytes sequester in the cerebral microvasculature of mice, which facilitates the passage of egressing parasites to the brain parenchyma (17). Additionally, *T. gondii* infects endothelial cells at the BBB (18, 19) and alters their barrier functions in a nondisruptive manner *in vitro* and *in vivo* (19–21).

The ability to modulate host cell signaling is likely a key factor in *T. gondii* infections. From its intercellular replicative niche, *T. gondii* targets host cell signal transduction and host gene transcription (22). The MYR translocon, primarily involving MYR1–4 proteins, is a specialized protein complex responsible for transporting parasite effector proteins from the parasite into the host cell cytoplasm (23, 24). MYR-dependent secretion allows *T. gondii* to manipulate host cellular functions, immune responses, and promote its survival. This system is likely also crucial for virulence as it enables the delivery of key effectors, like the NF-kB signaling modulator TEEGR (25), and other dense granule (GRA) proteins that facilitate intracellular persistence and immune evasion (22). Furthermore, MYR-independently secreted effectors, such as the NF-kB activating effector GRA15, also mediate important pro-inflammatory effects (26, 27).

The recent finding that infected phagocytes become sequestered within cortical capillaries raises key mechanistic questions about how *T. gondii* interacts with the cerebral endothelium during the early stages of CNS colonization (17). Here, we investigated the role of the CCL5/CCR5 axis in *T. gondii* dissemination. By exploring how this chemokine-chemokine receptor interaction influences the migratory behavior of infected DCs, we aimed to determine if *T. gondii* exploits this pathway to enhance its spread to the CNS.

## RESULTS

### *T. gondii* modulates CCL5 expression by endothelial cells via secreted effectors

**Fig 1:**
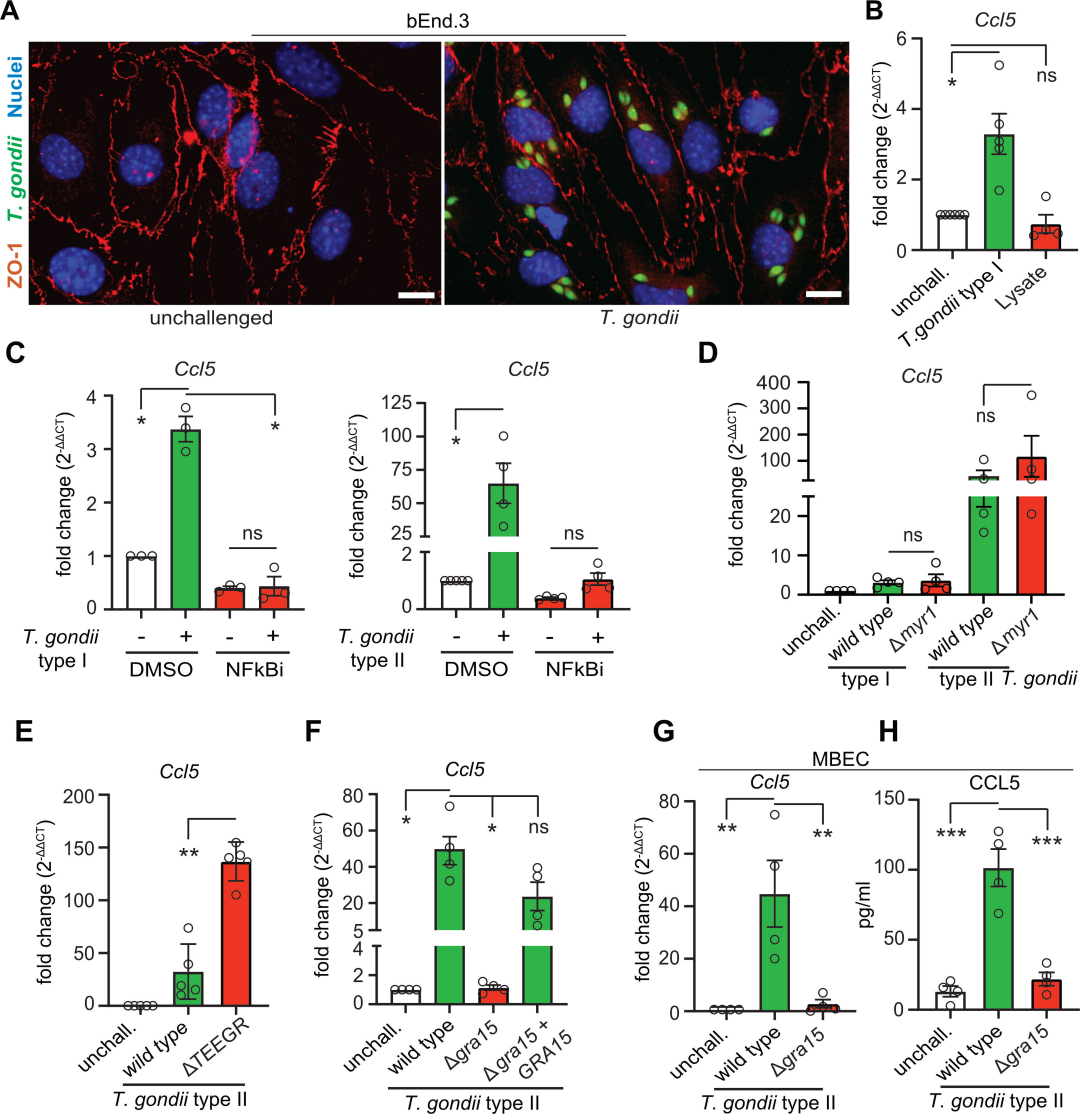
Effectors and cell signaling implicated in *Ccl5* expression and CCL5 secretion by endothelial cells upon challenge with *T. gondii.* (**A**) Representative micrographs of confluent bEnd.3 monolayers unchallenged or challenged with GFP-expressing T. gondii tachyzoites (RH-LDM, green) and stained for the tight junction protein ZO-1 (red), with DAPI-stained nuclei (blue). Scale bar = 20 μm. (**B–F**) Relative mRNA expression (quantitative polymerase chain reaction [qPCR]) of Ccl5 in confluent bEnd.3 cell monolayers challenged with freshly egressed *T. gondii* tachyzoites (indicated line/mutant, MOI 2, 24 h). The unchallenged condition indicates cells left untreated in the complete medium (CM) during the duration of the assay. (**B**) *T. gondii* type I (RH-LDM) and tachyzoite lysate (MOI 2-equivalent). (**C**) *T. gondii* type I (RH-LDM) and type II (PRU A7) in the presence of NF-kB inhibitors (NF-kBi) JSH-23 (9 μM) and TPCA-1 (3 μM), or dimethyl sulfoxide (DMSO) as a vehicle control. (**D**) *T. gondii* type I (RH, parental wild type) and MYR1-deficient mutant (Δ*myr1*); *T. gondii* type II (PRU Ku80, parental wild type) and MYR1-deficient mutant (Δ*myr1*). (**E**) *T. gondii* type II (PRU Ku80, parental wild type) and TEEGR-deficient mutant (Δ*TEEGR*). (**F**) *T. gondii* type II (PRU A7, parental wild type), GRA15-deficient mutant (Δ*gra15*), and reconstituted mutant (Δ*gra15*+GRA15). (**G**) Relative mRNA expression (qPCR) of Ccl5 in confluent primary MBEC monolayers challenged with *T. gondii* tachyzoites (PRU A7 wild type or Δ*gra15*, MOI 2, 24 h). (**H**) Abundance of CCL5 (pg/mlL) in supernatants from MBECs challenged as in panel (**G**) for 24 h, determined by ELISA. For each condition, data are from 3 to 5 independent experiments (n = 3–5). In graphs, means (± SEM) are indicated. qPCR data are displayed as fold change (2^-ΔΔCt^) in relation to the unchallenged condition. Statistical comparisons were performed with the mixed-effects model (**B, C**) and one-way analysis of variance (ANOVA), with Sidak's *post hoc* test (**D–H**) ;*P < 0.05, **P < 0.01, ***P < 0.001, ns: non significant.

The early infection of the cerebral endothelium by *T. gondii* during its process of CNS colonization (17–19) and the putative ability of cerebral endothelial cells to upregulate CCL5 upon inflammatory insults (6) motivated an investigation of this chemokine. To determine whether *T. gondii* infection impacts *Ccl5* expression, we challenged mouse brain endothelial cell (bEnd.3) monolayers with *T. gondii* (Fig. 1A) and observed that freshly egressed tachyzoites, but not multiplicity of infection (MOI)-equivalent tachyzoite lysates, induced a significant increase in *Ccl5* expression (Fig. 1B). Since NF-κB signaling is a known regulator of *Ccl5* expression (28), we inhibited NF-κB signaling pharmacologically. NF-κB inhibition abolished the infection-induced elevation of *Ccl5* expression, similarly for type I (RH) and type II (PRU) *T. gondii* strains (Fig. 1C). We also noted a remarkably superior relative induction of *Ccl5* expression by the type II strain (~3-fold elevation for type I vs ~60-fold for type II) (Fig. 1C). Next, we investigated the involvement of the parasite’s principal secretory system, MYR. Interestingly, challenge with MYR1-deficient parasites resulted in comparable (type I) or even a tendency for higher (type II) *Ccl5* expression (Fig. 1D), suggesting MYR1-independent and/or counter-regulatory effects. Indeed, deletion of the MYR-dependent effector TEEGR resulted in increased *Ccl5* expression (Fig. 1E), consistent with its attributed role of silencing a subset of NF-κB-regulated cytokines in fibroblasts and astrocytes (25). Given these findings, we hypothesized a role for the effector GRA15 because of its MYR1-independent NF-κB activation by type II strains and because the type I RH strain is known to express a nonfunctional form of GRA15 (26, 27). Consistent with this, deletion of GRA15 nearly abolished the elevated *Ccl5* expression, which was restored upon reconstitution of GRA15 (Fig. 1F), corroborating that GRA15 was crucial for *Ccl5* expression. Finally, in primary mouse brain endothelial cell (MBEC) monolayers, we confirmed that GRA15 induced transcriptional upregulation of *Ccl5* and increased secretion of CCL5 into the supernatant, as quantified by enzyme-linked immunosorbent assay (ELISA) (Fig. 1H). Together, these results show that GRA15 induces *Ccl5* expression and CCL5 secretion by endothelial cells via NF-κB activation, independent of MYR, while MYR-dependent TEEGR activity counteracts this response.

### CCL5 potentiates the motility of *T. gondii-*infected DCs in a CCR5-dependent fashion

**Fig 2:**
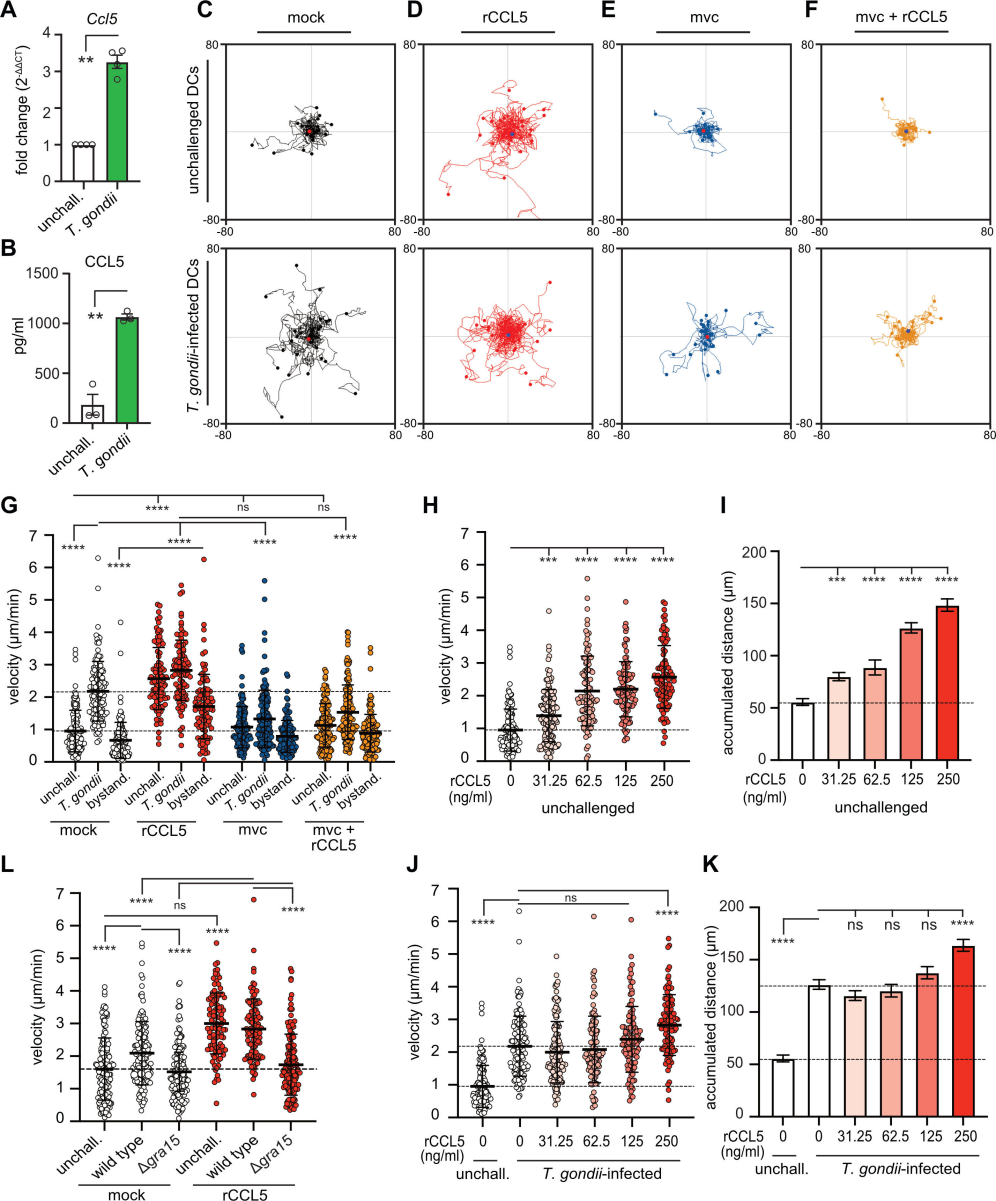
Effects of rCCL5 and CCR5 antagonism on the motility of *T. gondii*-challenged DCs. (**A**) Relative mRNA expression (mean ± SEM) of the *Ccl5* in primary DCs challenged with *T. gondii* tachyzoites (PRU A7, MOI 1, 24 h) related to unchallenged DCs. Data are from 4 independent experiments (*n* = 4). (**B**) Abundance of CCL5 (mean ± SEM) in supernatants from DCs challenged as in panel **A**, determined by ELISA. Data are from 3 independent experiments (*n* = 3). (**C–F**) Representative motility plots of unchallenged DCs (upper row) or *T. gondii*-infected DCs (lower row) that were mock-treated with CM (**C**), in the presence of rCCL5 in CM (**D**), treated with CCR5 antagonist mvc (**E**), or treated with rCCL5 after pretreatment with mvc (**F**). Scales are expressed in μm. (**G**) Velocities of unchallenged DCs, *T. gondii*-infected DCs, and bystander DCs subjected to mock treatment (CM), treatment with rCCL5 and mvc, or combined treatment mvc + rCCL5 in CM. Colored dots correspond to conditions in panels C–F and represent individual cells. Mean (±SD) is indicated, and dashed lines indicate mean velocities of mock-treated unchallenged and infected DCs, respectively. For each condition, 93–140 cells were analyzed from 3 to 4 independent experiments. (**H, I**) Graphs show velocities (**H**) and mean accumulated distances (**I**) of unchallenged DCs in the presence of rCCL5 at indicated concentrations. (**H**) Dots represent individual cells, and mean (±SD) is indicated. (**I**) Bars represent mean (±SEM) accumulated distances. Dashed lines indicate mean velocity (**H**) or mean accumulated migrated distance (**I**), respectively, of cells in the absence of rCCL5. For each condition, 94–132 cells were analyzed from 3 to 4 independent experiments. (**J, K**) Velocities (**J**) and mean accumulated distances (**K**) of *T. gondii*-infected DCs in the presence of rCCL5 as in panel **H and I**. (**J**) Dots represent individual cells, and mean (±SD) is indicated. (**K**) Bars represent mean (±SEM) accumulated distances. Dashed lines indicate mean velocity (**J**) or mean accumulated migrated distance (**K**), respectively, of mock-treated unchallenged and infected DCs. For each condition, 96–134 cells were analyzed from 3 to 4 independent experiments. (**L**) Velocities of unchallenged DCs, DCs infected with *T. gondii* wild type (PRU A7) or GRA15 mutant (PRUΔ*gra15*), in CM (mock) or with added rCCL5. Mean (±SD) is indicated, and the dashed line indicates the mean velocity of unchallenged mock-treated cells. For each condition, 100–151 cells were analyzed from 3 to 4 independent experiments. For all experiments, rCCL5 was used at 250 ng/mL, unless differently indicated, and mvc at 10 μM. Statistical comparisons were performed with Student’s *t*-test (**A, B**) and one-way ANOVA, with Sidak’s *post hoc* test (**G–L**); **P < 0.01, ***P < 0.001, ****P < 0.0001, ns: nonsignificant.

The robust CCL5 responses by endothelial cells following *T. gondii* (type II) challenge (Fig. 1), together with previous findings implicating infected DCs in parasite dissemination (13, 17), prompted us to explore the potential role of the CCL5/CCR5 axis in the hypermigratory phenotype of infected DCs (14). Notably, a key *in vitro* characteristic of parasitized DCs is their random-directional hypermotility in the absence of a chemokine gradient (15, 29). We first confirmed that *T. gondii* exposure increased *Ccl5* transcription in DCs (Fig. 2A), which was accompanied by a strongly elevated CCL5 secretion into the cell supernatants (Fig. 2B). To assess the impact of elevated CCL5 on the migratory behavior of both unchallenged and *T. gondii*-infected DCs, we measured DC motility *in vitro* in the presence of recombinant CCL5 (rCCL5) or the CCR5-selective antagonist Maraviroc (mvc) (30, 31) (Fig. 2C through G). Consistent with previous reports (14, 15, 29), challenge with *T. gondii* increased the velocities of infected DCs, without velocity increase in non-infected *T. gondii*-challenged DCs, defined as *bystander* DCs (Fig. 2C and G, black). In contrast, treatment with rCCL5 significantly enhanced motility in both unchallenged and infected DCs and also elevated the motility of bystander DCs (Fig. 2D and G, red). CCR5 antagonism with mvc reduced velocities for all conditions (Fig. 2E and G, blue), and subsequent rCCL5 treatment of mvc-treated cells did not restore velocities (Fig. 2F and G, orange). Together, these results indicate that CCL5/CCR5 signaling modulates hypermotility in *T. gondii*-infected DCs (14). Dose-response experiments further corroborated that rCCL5 increased both velocities (Fig. 2H) and migration distances (Fig. 2I) of DCs in a concentration-dependent manner, with infected DCs reaching maximal velocities at lower concentrations than unchallenged DCs (Fig. 2J and K). Finally, deletion of GRA15 resulted in reduced velocities of infected DCs (Fig. 2L). Notably, this phenotype of Δ*gra15-*infected DCs was not restored by rCCL5, unlike the responses observed in unchallenged DCs (Fig. 2L), pointing to additional, GRA15-dependent alterations in CCR5 expression or activity. Collectively, these findings show that GRA15-mediated activation of the CCL5/CCR5 axis contributes to the hypermotility phenotype of *T. gondii*-infected DCs, likely through both chemokinetic and chemotactic effects.

### *T. gondii*-infected DCs perform chemotaxis toward CCL5, potentiating transmigration across endothelial monolayers *in vitro*

**Fig 3:**
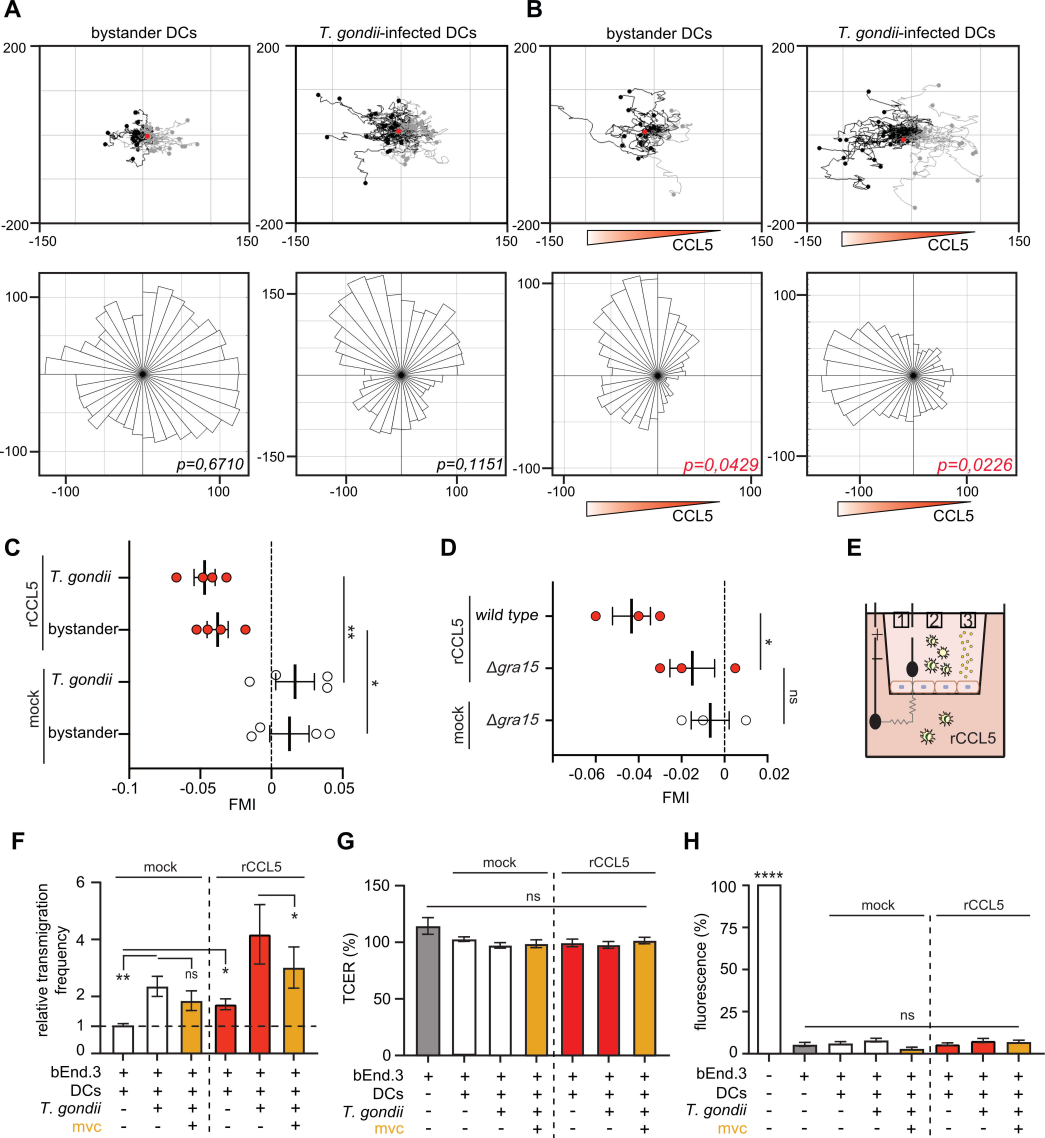
Chemotactic responses and transmigration of *T. gondii*-challenged DCs toward rCCL5. (**A, B**) Representative motility plots of *T. gondii* (PRU A7)-infected or bystander DCs in a collagen matrix with CM (**A**) or with a rCCL5 gradient (**B**), as detailed in Materials and Methods. Scales are expressed in μm. Tracks of cells migrating to the left side from their initial position are shown in black, and the opposite in gray. The red dot represents the center of mass. For each condition, a rose plot diagram depicts the chemotactic tendency of the cell population. *P*-values indicate the chemotaxis effect compared to hypothetical uniformity of a circular distribution of cell endpoints (Rayleigh test as described in Methods). Plots depict 60 tracked cells per condition from 1 representative experiment. (**C**) Mean forward migration index (FMI) for *T. gondii*-infected or bystander DCs in a collagen matrix with CM (mock) or with a rCCL5 gradient, as described under Materials and Methods. Data are from 168 to 220 analyzed cells per condition from 4 independent experiments. (**D**) Mean FMI for DCs infected with *T. gondii* wild type II (PRU A7) or GRA15 mutant (PRUΔ*gra15*), in CM (mock) or with a rCCL5 gradient. Data are from 77 to 89 analyzed cells per condition from 3 independent experiments. (**E**) Schematic representation of the experimental setup in a transwell filter system with polarized endothelial monolayers grown in the upper compartment of the culture insert: (1) TCER, (2) DCs challenged with *T. gondii* tachyzoites added to the upper compartment, and (3) FITC-dextran (3 kDa) fluorescent permeability tracer added to the upper compartment and measured in the lower compartment, as detailed under Materials and Methods. (**F**) Relative transmigration of DCs challenged with *T. gondii* tachyzoites across polarized bEnd.3 cell monolayers in the presence of rCCL5 (250 ng/mL) added to the lower compartment. When indicated, DCs were pretreated with mvc (10 μM) 1 h prior to the start of the assay. Transmigration frequency indicates the ratio of transmigrated DCs (counted in a lower chamber) related to the number of DCs initially added to the upper chamber. Shown as fold change in relation to the control condition (unchallenged mock-treated DCs). (**G**) TCER of bEnd.3 cell monolayers after DCs transmigration. Data are shown as % TCER values (Ω•cm^2^) relative to TCER values at the initiation of the assay (100%), as detailed in Materials and Methods. (**H**) Permeability to FITC-dextran (3 kDa) of bEnd.3 cell monolayers after DCs transmigration. Signal, measured as arbitrary fluorescence units (AU), from leaked FITC-dextran in the lower compartment was recorded at the end of the transmigration assay and related to the signal of the culture insert in the absence of a polarized monolayer (100%). Data are from 3 (**D**), 4 (**C**), or 6 (**E–G**) independent experiments. Means (±SEM) are indicated. Statistical comparisons were performed with one-way ANOVA, with Sidak’s *post hoc* test (**C, D**) and mixed-effects model (**F–H**) ;*P < 0.05; **P < 0.01, ****P < 0.0001, ns: nonsignificant.

To investigate whether the CCL5-/CCR5-dependent increase in motility of infected DCs involved chemotaxis, we exposed cells to rCCL5 gradients in chemotaxis chambers (16). In the absence of rCCL5, infected DCs exhibited random-directional motility that was superior to that of bystander DCs, as expected (Fig. 3A). However, in the presence of a rCCL5 gradient, both infected and bystander DCs migrated directionally toward rCCL5 (Fig. 3B and C). Moreover, deletion of GRA15 diminished chemotaxis toward rCCL5 (Fig. 3D), in line with effects on hypermotility (Fig. 2). Next, we assessed whether CCL5 also affected the translocation of infected DCs across polarized endothelial monolayers using a transwell assay (32) (Fig. 3E). In the presence of rCCL5 in the lower transwell compartment, *T. gondii*-infected DCs added to the upper compartment exhibited a higher frequency of transmigration, and bystander DCs also showed a smaller but significant increase (Fig. 3F, red bars). Notably, CCR5 antagonism significantly reduced the transmigration frequency of *T. gondii*-infected DCs in the presence of rCCL5, whereas only a nonsignificant reduction was observed in its absence (Fig. 3F, orange bars). To ensure the integrity of the endothelial barrier during the assay, we measured transcellular electrical resistance (TCER) and assessed impermeability to a fluorescent tracer (FITC-dextran). Both parameters were stable throughout the assay (Fig. 3G and H), confirming that barrier integrity was maintained. Collectively, these results indicate that CCL5 contributes to the migratory behavior of *T. gondii*-infected DCs, promoting chemotactic responses and enhancing their transmigration across polarized endothelial monolayers.

### *T. gondii* infection induces CCL5 production and rCCL5 triggers early cerebral microvascular activation

**Fig 4:**
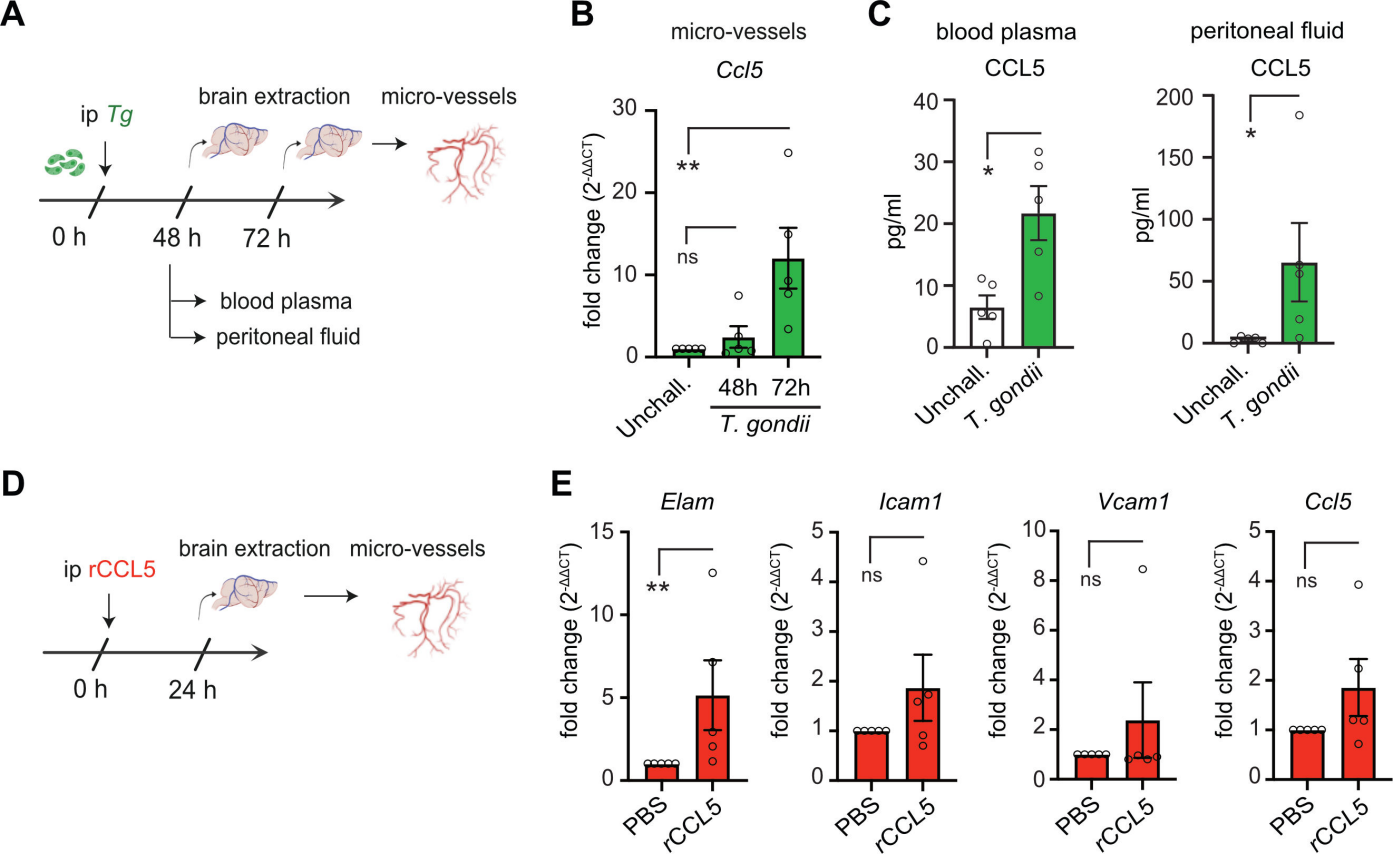
CCL5 responses and inflammatory responses by the cerebral microvasculature upon challenges with *T. gondii* and rCCL5. (**A**) Experimental setup. Mice were inoculated ip with 3 × 10^5^ cfu of freshly egressed tachyzoites (ME49-RFP, ip*Tg*) or control medium. Brains and fluids were collected, and cortical microvessels were purified as detailed in Materials and Methods, at indicated time points. (**B**) Relative mRNA expression (qPCR) of *Ccl5* in purified brain microvessels at indicated time points in mice treated as in panel **A** (*n* = 5 mice). (**C**) Abundance of CCL5 (pg/mL) in blood plasma and peritoneal lavage, determined by ELISA, in mice treated as in panel **A** (*n* = 5 mice). (**D**) Experimental setup. Mice were treated ip with rCCL5 (1 µg) or mock treated, brains were collected, and microvessels were purified after 24 h. (**E**) Relative mRNA expressions (qPCR) of *Elam1* (E-selectin), *Icam1* (ICAM-1), *Vcam1* (VCAM-1), and *Ccl5* in brain microvessels from mice treated as in panel **D** (*n* = 5 mice). All data are displayed as mean (±SEM) and are from 2 (**C**) or 3 (**B, E**) independent experiments. qPCR data are displayed as fold change (2^-ΔΔCt^) in relation to the mock-treated condition. Statistical comparisons were performed with one-way ANOVA, Sidak’s *post hoc* test (**B**), and two-tailed Mann-Whitney *U*-test (**C, E**), *P < 0.05, **P < 0.01, ns: nonsignificant.

The *in vitro* findings of CCL5 production by endothelial cells upon *T. gondii* challenge and the enhanced migratory behavior of infected DCs led us to explore the role of CCL5 *in vivo*. In mice inoculated intraperitoneally (ip) with *T. gondii*, we observed a rapid (72 h) increase in *Ccl5* expression in purified cortical microvessels (Fig. 4A and B). Consistently, elevated levels of CCL5 were also detected in blood plasma and peritoneal fluid by 48 h, as measured by ELISA (Fig. 4C). Finally, we sought to determine the earliest time point of cerebral microvascular activation following ip challenge with rCCL5 (Fig. 4D). At 24 h post-challenge, transcriptional upregulation of *Elam* (E-selectin) was detected, whereas *Icam1*, *Vcam1*, and *Ccl5* expressions exhibited nonsignificant elevations in cortical microvessels (Fig. 4E). Collectively, these findings indicate a rapid inflammatory response within the cerebral microvasculature, corroborating and extending recent observations (17). We conclude that *T. gondii* infection induces CCL5 production and that rCCL5 promotes inflammatory activation of the cerebral microvasculature. Together, these data prompted us to investigate the potential role of CCL5/CCR5 signaling in the sequestration of infected DCs in brain capillaries (17).

### Infection-induced CCL5 elevates microvascular sequestration of infected DCs and cerebral parasite loads

**Fig 5:**
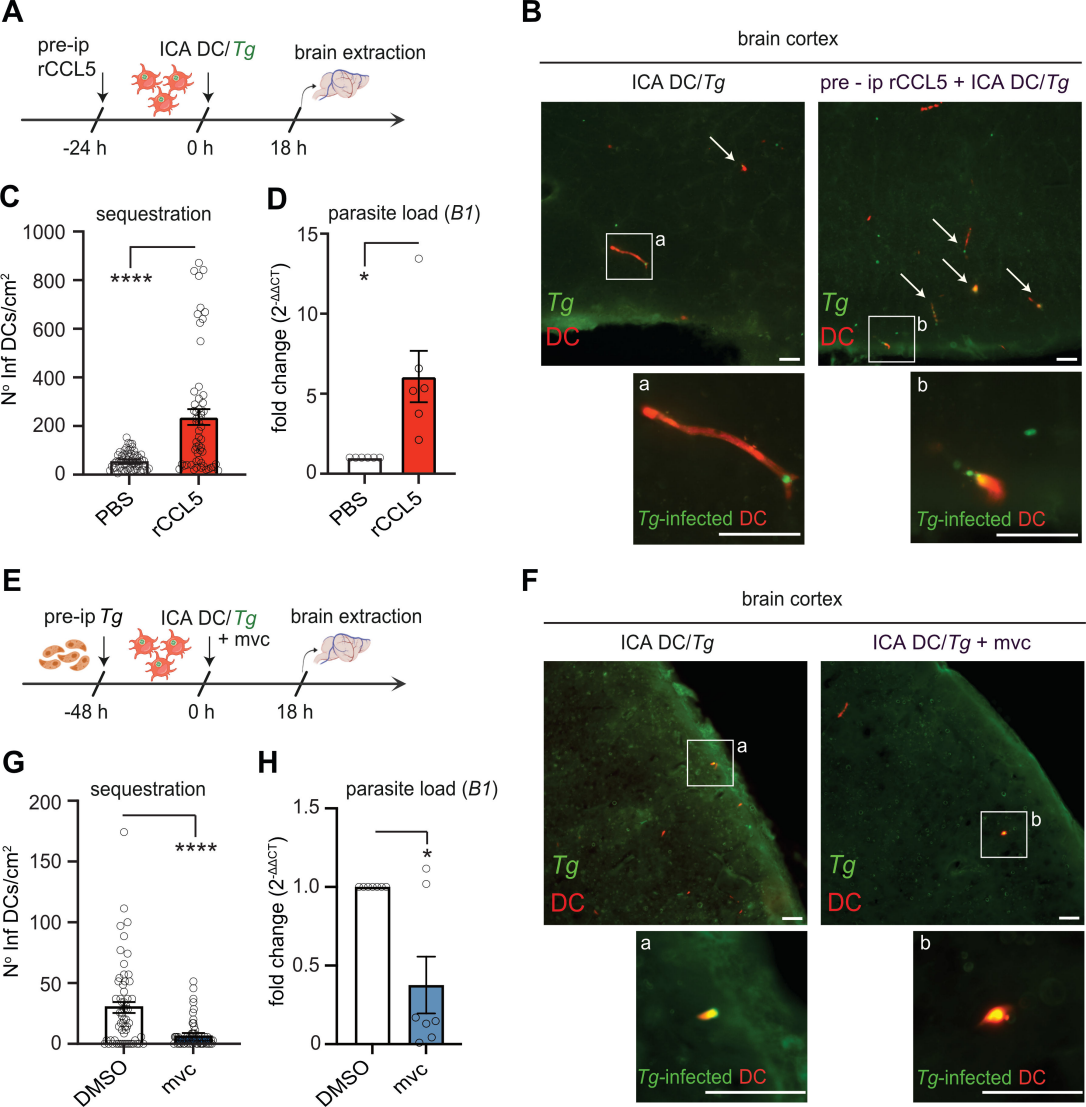
Impact of rCCL5 and CCR5 antagonism on the sequestration of *T. gondii*-infected DCs and on cerebral parasite loads. (**A**) Experimental setup. Mice were pretreated ip (pre-ip) with rCCL5 (1 µg) or mock-treated (PBS). CMTMR-labeled DCs were challenged with *T. gondii* (PRU-GFP, MOI 2) to obtain an infection frequency of ~50%. Challenged DCs (12 × 10^6^ DCs*/*~6 × 10^6^ cfu *Tg*) were then directly inoculated into the brain circulation via the ICA, as detailed in Materials and Methods. Brains were collected at 18 h post-inoculation. (**B**) Representative micrographs of *T. gondii*-infected DCs (CMTMR^+^ GFP^+^) sequestered in the cortical tissues of mice pretreated and inoculated as in panel **A**. Arrows indicate *T. gondii*-infected DCs (CMTMR^+^ and GFP^+^), and insets (**A, B**) show magnifications. Scale bars: 50 µm. (**C**) Graph shows the absolute numbers (mean ± SEM) of infected DCs (CMTMR^+^ and GFP^+^) per cm^2^ cortical tissue for rCCL5-treated and mock-treated mice. Data are from 58 to 60 cortical sections per condition from 3 independent experiments (*n* = 6 mice). (**D**) Relative expression (qPCR) of *T. gondii* TgB1 gene in the brain tissue of mice pretreated and inoculated as in panel **A** (*n* = 6 mice). (**E**) Experimental setup. Mice were pre-inoculated ip with 3 × 10^5^ cfu type II ME49-RFP tachyzoites. CMTMR-labeled DCs were challenged with *T. gondii* (PRU-GFP, MOI 2) to obtain an infection frequency of ~50%. Challenged DCs (8 × 10^6^ DCs*/*~4 × 10^6^ cfu *Tg*) were directly inoculated into the brain circulation via the ICA, jointly with mvc (10 µM) in the cell suspension) or mock treatment (DMSO). Brains were collected at 18 h post-inoculation. (**F**) Representative micrographs show infected DCs (CMTMR^+^ and GFP^+^) in the brain cortex of mice pretreated as in panel **E**. Insets (**A, B**) show magnifications of the infected DCs (CMTMR^+^ and GFP^+^). Scale bars: 50 µm. (**G**) Graph shows the absolute numbers (mean ± SEM) of infected DCs (CMTMR^+^ and GFP^+^) per cm^2^ cortical tissue for mvc-treated and mock-treated mice. Data are from 60 cortical sections per condition (*n* = 6 mice). (**H**) Relative expression (qPCR) of the *T. gondii* TgB1 gene in the brain tissue of mice inoculated as in panel **E** (*n* = 7 mice). Data are from 3 (**C, D, G**) or 4 (**H**) independent experiments. Means (±SEM) are indicated. Statistical comparisons were performed with two-tailed Mann-Whitney *U*-test, *P < 0.05, ****P < 0.0001, ns: nonsignificant.

To determine whether early CCL5 responses affect the sequestration of parasitized leukocytes, we introduced infected DCs into the cerebral circulation of mice via intracarotid artery (ICA) injection (17) (Fig. 5A). Notably, pretreatment with rCCL5 ip markedly enhanced the sequestration of infected DCs within the cerebral microvasculature by 18 h post-inoculation (Fig. 5B and C), resulting in increased cerebral parasite burdens (Fig. 5D). Next, given that *T. gondii* infection rapidly elicited robust CCL5 responses in the cerebral microvasculature and blood circulation (Fig. 4), we pre-infected animals with *T. gondii* to establish an inflammatory baseline (17) prior to CCR5 antagonism (Fig. 5E). Importantly, administration of the CCR5 antagonist mvc significantly reduced the sequestration of infected DCs (Fig. 5F and G) and reduced parasite loads (Fig. 5H). Collectively, these findings show that CCL5/CCR5 signaling promotes the sequestration of parasitized DCs, thereby contributing to elevated cerebral parasite burdens early during infection. Building on prior evidence showing reduced sequestration by GRA15-deficient parasites (17), our data establish a mechanistic link between GRA15 and the CCL5/CCR5 axis in elevating infected DC sequestration, and thereby parasite loads.

## DISCUSSION

This study identifies a role for CCL5 in facilitating the dissemination of *T. gondii* at the BBB, highlighting its contribution to the parasite’s colonization of the CNS.

Our findings demonstrate that in response to CCL5, *T. gondii*-infected DCs exhibit enhanced motility, chemotactic activity, and transendothelial migration *in vitro*. The hypermigratory phenotype of parasitized phagocytes engages several host cell signaling pathways, including the CCL19/CCR7 axis, as well as various parasite effectors (14, 33, 34). In this study, we add that activation of the CCL5/CCR5 signaling axis also contributes to this response. Notably, infected DCs displayed elevated random directional motility—hypermotility (14)—in response to rCCL5 even under nongradient conditions. This suggests a chemokinetic or haptokinetic response to CCL5, in contrast to the lack of a comparable response following CCL19/CCR7 activation (16). Furthermore, this migratory response was enhanced in a rCCL5 gradient, resulting in increased chemotaxis and transendothelial migration across polarized monolayers. Inhibition of these migratory phenotypes by the CCR5 antagonist mvc supports the role of CCL5 in mediating chemotactic signaling. Although the various *in vitro* assays used may differentially emphasize the contribution of CCL5/CCR5 signaling, the consistent detection of measurable effects across all assays supports its overall role in the hypermigratory phenotype. Furthermore, no significant alterations were detected in endothelial permeability parameters (TCER and FITC–dextran assays), indicating that neither rCCL5 stimulation nor CCR5 antagonism compromised cellular barrier integrity, despite facilitating the transmigration of parasitized DCs. Collectively, these *in vitro* findings suggest that the CCL5/CCR5 signaling axis contributes to the migratory activation of *T. gondii*-infected DCs, an *in vitro* phenotype previously linked to systemic dissemination to peripheral organs in mice (13, 15).

The data establish that the effector GRA15 (26), secreted independently of the MYR translocon (23), plays a central role in driving CCL5 upregulation in endothelial cells upon *T. gondii* (type II, ME49, PRU) infection, primarily through NF-κB activation. In contrast, the MYR-dependent effector TEEGR acted as a negative regulator of CCL5 expression, likely by antagonizing NF-κB signaling (25). Together, these findings indicate that *T. gondii* tightly regulates CCL5 expression, with significant implications for the recruitment of parasitized leukocytes to the BBB endothelium. Given that GRA15 activates NF-κB (26) and CCL5 signaling can also trigger NF-κB activation in endothelial cells (35), our results suggest that GRA15-induced CCL5 contributes, at least in part, to the amplification of endothelial activation, specifically through the expression of E-selectin, ICAM-1, and VCAM-1. Furthermore, deletion of GRA15 reduced infected DC hypermotility *in vitro*, an effect that was unexpectedly not rescued by rCCL5. This aligns with the decreased chemotactic response to rCCL5 observed in this mutant. Accordingly, DCs infected with the GRA15 mutant exhibited reduced transmigration across the polarized endothelium (32). Assuming that pro-inflammatory GRA15 signaling impacts CCR5 expression or associated signal transduction, probably via NF-κB activation and subject to potential counter-regulation via TEEGR, a comprehensive analysis of the CCL5/CCR5/NF-κB regulatory circuit (3), in the context of additional pro-inflammatory signals (36), is warranted. Besides inducing pro-inflammatory CCL5, GRA15 also directly upregulates ICAM-1 of infected cells (32), which impacts phagocyte-endothelium interactions (37) and, specifically, ICAM-1-mediated sequestration of infected phagocytes (17). Notably, the elevated sequestration frequencies of infected phagocytes characteristic of type II ME49 and PRU strains (17), relative to the type I RH strain that lacks a functional GRA15 (26), are likely attributable, at least in part, to enhanced CCL5. Based on the experimental findings of (i) dramatically increased sequestration of parasitized DCs and elevated parasite loads following rCCL5 treatment, (ii) reduced sequestration and reduced parasite loads upon CCR5 antagonism, and (iii) enhanced sequestration driven by GRA15 (17), we propose that GRA15 amplifies NF-κB-mediated signaling, thereby promoting CCL5-/CCR5-dependent leukocyte homing to the cerebral microvasculature. Nonetheless, chemokine-chemokine receptor signaling is characterized by context-dependent redundancy (38). Although CCL5 exhibits its highest affinity for CCR5, it can also bind to CCR1 and CCR3 (4). While additional contributions from CCR1 and CCR3 to the observed phenotypes cannot be entirely ruled out, they are likely minimal given the potent inhibition achieved both *in vitro* and *in vivo* by the highly CCR5-selective antagonist mvc (30, 31). Moreover, the immediate effects of CCR5 agonism and antagonism in our experiments likely minimized the compensatory adaptive mechanisms reported in CCR5 mutant mouse models (38, 39). Additionally, DCs maintain CCR5 expression upon challenge with *T. gondii* (15, 40) and the *T. gondii* effector cyclophilin-18, which binds CCR5, may hypothetically contribute to leukocyte dissemination through both CCR5-dependent (41) and CCR5-independent (42) mechanisms, although this requires experimental validation. Finally, recent findings that several *T. gondii* effectors, including GRA15, GRA24, and GRA28, co-operatively promote CCR7-mediated migration of infected phagocytes to peripheral organs raise questions about the role of the CCL19, −21/CCR7 axis (16, 27). However, direct evidence for endothelial CCL19 and 21 in mediating immune cell trafficking to the CNS in the absence of neuroinflammation remains lacking (43, 44) and warrants further investigation.

The unexpectedly rapid elevation of *Ccl5* expression in the brain microvasculature following intraperitoneal *T. gondii* challenge suggests a prompt activation response that likely primes the cerebral endothelium for the recruitment of infected phagocytes. This swift CCL5 upregulation, also evident in blood, parallels the increased expression of cell adhesion molecules (CAMs) in the cerebral microvascular endothelium, notably E-selectin, ICAM-1, and VCAM-1 (17, 19), and precedes the markedly elevated systemic cytokine responses observed later during infection (45). Consistent with these findings, elevated CCL5 protein levels have been detected in mouse brain during *T. gondii* infection (46), as well as in the serum of infants with congenital toxoplasmosis (47) and pregnant women affected by toxoplasmosis (48).

Building on the recent finding that GRA15 promotes the sequestration of infected phagocytes in cortical capillaries (17), the present data identify a CCL5-driven molecular mechanism through which *T. gondii* enhances leukocyte homing to the CNS, thereby facilitating the sequestration of parasitized leukocytes and brain colonization by *T. gondii*. In line with this, CCL5 is known to localize to endothelial surfaces (49) and has been associated with enhanced leukocyte adhesion to the endothelium in both *Herpes simplex* encephalitis and autoimmune encephalomyelitis (50, 51). Notably, the CCL5/CCR5 axis appears to be co-opted for microbial immune evasion, with anti-apoptotic effects on influenza virus-infected macrophages (52) and as a target for *Staphylococcus aureus* cytotoxins (53). In toxoplasmosis, our findings indicate that CCL5/CCR5 signaling functions as a homing cue that elevates the sequestration of parasitized phagocytes in cerebral vasculature and, ultimately, influences cerebral parasite burden at the early stages of CNS colonization. While the precise roles of CCL5 in the pathogenesis of human or murine toxoplasmosis remain unexplored (46–48), its ligand CCR5 has been implicated in regulating natural killer cell responses in mice (54) and in promoting pro-inflammatory activity in DCs, particularly through the induction of interleukin 12, a key cytokine in the immune response to *T. gondii* in mice (55, 56). It is therefore plausible that GRA15 promotes, while TEEGR attenuates, these pro-inflammatory pathways, striking a balance in the cerebral microenvironment that facilitates effective leukocyte homing without triggering excessive inflammation. Interestingly, the manner in which *T. gondii* manipulates the CCL5/CCR5 axis to facilitate dissemination mirrors to some extent the hijacking of this same chemokine pathway in cancer metastasis (5), including immunomodulatory effects that favor tumor growth (57). Finally, given that CCL5/CCR5 signaling can activate microglia and astrocytes (7–9), our findings raise intriguing questions about the putative roles of the *T. gondii*-induced CCL5/CCR5 axis in initiating or driving neuroinflammation early during primary infection.

Together, the data reveal that *T. gondii* hijacks chemotactic signaling in a refined manner, specifically through GRA15-induced CCL5 responses during primary infection. This may play a critical role in the early sequestration of infected phagocytes within the brain microvasculature, thereby contributing to the early colonization of the CNS by *T. gondii*. We propose that the CCL5/CCR5 axis is finely regulated in infected cells by the opposing actions of effectors GRA15 and TEEGR on NF-κB signaling. Regardless of the route of inoculation, accumulating evidence suggests that brain colonization by *T. gondii* occurs through relatively few invasion events compared to total invasion events in other organs (19) and begins earlier than previously estimated (17). This stealthy pattern of invasion aligns with the typically mild or asymptomatic presentation of natural primary infection in humans and rodents (10, 11). Yet, the cues that determine whether parasitized phagocytes sequester or transmigrate remain to be determined. Infected phagocytes preferentially undergo sequestration during the very early stages of infection (17) and may, hypothetically, later transition toward transmigration in response to inflammatory or chemotactic signals originating from the brain parenchyma; however, this remains to be demonstrated. Notably, microglia and astrocytes are key producers of CCL5 in response to inflammation in the CNS (7).

The emerging picture suggests that *T. gondii* employs a multifaceted, but subtle, strategy to overcome the restrictiveness of the BBB, based on the absence of measurable barrier disruption, microhemorrhage, or significant inflammatory cell infiltration early during colonization (17, 19). In mouse models, it is likely important to distinguish this early, relatively silent, colonization phase from the later inflammatory processes that occur once systemic inflammation has set in during infection (58, 59), which later implicate, among others, CCL2 responses and generalized NF-kB activation by infiltrating leukocytes (60). Specifically, early GRA15-/CCL5-mediated leukocyte sequestration likely occurs alongside GRA24-induced endothelial secretion of the anti-inflammatory tissue inhibitor of metalloproteinases 1 (TIMP1) (21), as well as the dysregulation of focal adhesion kinase (FAK/PTK2), which destabilizes endothelial tight junctions (19, 20). Hypothetically, these mechanisms may collectively facilitate *T. gondii* colonization of the CNS by orchestrating a balance of effects over time: enhancing the homing of infected leukocytes to the cerebral vasculature, promoting endothelial cell invasion and tachyzoite transmigration across the BBB, and simultaneously preserving immune control while limiting excessive, host-detrimental inflammation. Given the successful targeting of the CCL5/CCR5 axis in HIV infection, certain hematological malignancies, and solid tumors (5, 31), our findings highlight the potential for developing future preventive or adjunctive therapies against severe disseminated toxoplasmosis.

## MATERIALS AND METHODS

### Mice

All experiments were performed using male and female C57BL/6NCrl mice (strain code 027, Charles River), aged 4 to 10 weeks, housed in a ventilated facility, provided with unrestricted access to tap water and food, and kept under a 12-h light/dark cycle at a temperature of 20°C–22°C.

### Primary DCs

Murine bone marrow-derived DCs were generated as previously described (15). Briefly, cells from bone marrow of 6- to 10-week-old male or female C57BL/6NCrl mice (Charles River) were cultured in RPMI 1640 (VWR) with 10% fetal bovine serum (FBS; HyClone), gentamicin (20 μg/mL; Sigma-Aldrich), glutamine (2mM) and HEPES (0.01 M), and supplemented with 10 ng/mL recombinant mouse GM-CSF (Peprotech), defined as CM. Loosely adherent cells (DCs) were harvested after 6 or 8 days of maturation.

### Isolation of cerebral microvessels and MBECs

Brain microvessels were isolated as previously described (19). Briefly, brain homogenates (23-gauge needle) were diluted 1:1 (vol/vol) with 30% dextran solution (MW 70.000; Sigma-Aldrich, Cat# 31390-25) and centrifuged at 10,000 × *g* for 15 min at 4°C. The myelin layer was discarded and the pellet resuspended in PBS. The suspension was passed through a 40-µm cell strainer and retrieved vessel fragments washed with PBS. Finally, the cell strainer was back-flushed with PBS and the microvessels pelleted by centrifugation at 4,500 × *g* for 30 min at 4°C. Isolated micro-vessels were either used in assays or to derive MBECs.

For *in vitro* culture of MBECs, microvessels were incubated with 1 mg/mL collagenase IV (Gibco) and 200 U/mL of DNase I (Roche) dissolved in HBSS (Ca^2+^ and Mg^2+^) for 1.5–2 h at 37°C. Digested microvessels were washed and seeded in 12-well plates pre-coated with 1% gelatin (Gibco) in EBM-2/EGM-2 medium (Lonza) with 4 μg/mL puromycin (Santa Cruz). Purity of MBECs was determined by CD31 expression and ZO-1 expression, as detailed in 20. MBECs were challenged with freshly egressed *T. gondii* tachyzoites and processed for PCR.

### Cell lines, parasite culture, and infection challenges

Human foreskin fibroblasts (HFF-1 SCRC-1041, American Type Culture Collection) and bEnd.3 cells (CRL-2299, American Type Culture Collection) were cultured in Dulbecco’s modified Eagle’s medium, high glucose (DMEM; HyClone), with 10% FBS (HyClone), 20 μg/mL gentamicin (Sigma-Aldrich) and 2 mM L-glutamine at 37°C/5% CO_2_. All cell cultures used were periodically tested for mycoplasma and found to be negative.

*T. gondii* lines used are listed in Table S1. *T. gondii* tachyzoites were maintained by serial 48-h passages in HFF-1 monolayers. bEnd.3 cells, MBECs, and DCs were challenged with freshly egressed *T. gondii* tachyzoites at indicated MOIs, to reach an infection frequency of 50%–80%. An infected cell was defined as a cell associated with GFP signal or containing a vacuole with replicating GFP^+^ *T. gondii. T. gondii*-challenged GFP^-^ (uninfected) cells were defined as bystander cells.

### Reagents and ELISA

Reagents used are listed in Table S1. *T. gondii* lysates were generated from freshly egressed *T. gondii* tachyzoites by repeated freeze-thaw cycles. Supernatants from bEnd.3 monolayers, MBECs, and DCs challenged with freshly egressed *T. gondii* tachyzoites, plasma, or peritoneal fluid were analyzed using ELISA (CCL5, Invitrogen) according to the manufacturer’s instructions.

### Motility assays

Motility assays were performed as previously described (40). Briefly, DCs were cultured in 96-well plates in CM with or without freshly egressed *T. gondii*-tachyzoites (PRU A7-GFP or PRUΔ*gra15-*GFP, MOI 3, 4 h). When indicated, cells were treated with mvc (10 μM) for the last hour of the infection challenge. Cell suspensions in CM were embedded in bovine collagen type I (1 mg/mL, Sigma), rCCL5 (250 ng/mL) was added, and live cell imaging was performed for 1 h, 1 frame/min at 10× magnification (Z1 Observer with Zen 3 Blue, Zeiss). Time-lapse images were consolidated into stacks, and motility data were obtained from 30 to 35 cells per condition (Manual Tracking, ImageJ) and analyzed with Chemotaxis and Migration tool (Ibidi). Infected cells were identified by GFP^+^ signal.

### Chemotaxis assays

DCs were challenged with freshly egressed *T. gondii*-tachyzoites (PRU A7-GFP or PRUΔ*gra15-*GFP, MOI 1.5, 4 h), then resuspended in CM with bovine collagen type I (1 mg/mL), and seeded into ibiTreat μ-slide chemotaxis chambers (Ibidi, Martinsried, Germany). Collagen was allowed to polymerize for 30 min, and then media and rCCL5 (250 ng/mL) were added as indicated and according to the manufacturer’s instructions. Live cell imaging was performed for 2–4 h, 1–2 frames/min at 10× magnification (Z1 Observer with Zen 3 Blue, Zeiss). Time-lapse images were consolidated into stacks, and motility data were obtained typically from 30 to 60 cells per condition (Manual Tracking, ImageJ) and analyzed with the Chemotaxis and Migration tool (ImageJ). Infected cells were identified by GFP^+^ signal. The FMI, as a measure of cell migration in a direction along the *x*-axis parallel to the gradient, was calculated for each condition with the Chemotaxis and Migration tool (ImageJ). The Rayleigh test was used to test the uniformity of a circular distribution of cell endpoints.

### Polarization of endothelial cell monolayers and permeability assays

bEnd.3 cells cultured to 80% confluence were seeded onto culture inserts (8-μm pore size; Corning Transwell) and grown for 5 days until they reached polarization, defined as a stable TCER for 2 days above 200 Ω • cm^2^. TCER was measured before and after transmigration using an Ohmmeter (Millipore, Bedford, MA) and calculated with the following formula: unit area resistance (TCER) = resistance (Ω) • effective membrane area (cm^2^). Values are shown as percentages of TCER related to TCER prior to transmigration. For evaluation of cell monolayer permeability following transmigration, FITC-dextran (3 kDa; Life tech) was added to the upper compartment of the culture insert at a concentration of 12.5 μg/mL for 90 min. The medium was collected from the lower compartment, and fluorescence was measured in a fluorometer (EnSpire Multimode Plate Reader, PerkinElmer) at 485-nm excitation and 520-nm emission.

### Transmigration assays

Transmigration assay was performed as previously described (37), with modifications. DCs were challenged with freshly egressed *T. gondii* tachyzoites for 4 h with MOI 2, resulting in 60%–70% infection frequency or left unchallenged. When indicated, DCs were treated with mvc (10 μM) for 1 h prior to transmigration. Murine recombinant CCL5 (rCCL5, 250 ng/mL, Peprotech) was added to the lower compartment of culture inserts with pre-cultured polarized monolayers of bEnd.3 cells 30 min prior to the start of the assay. DCs were then transferred to the upper compartment of the culture inserts and allowed to transmigrate for 16 h. Transmigrated DCs were incubated on ice for 15 min to disassociate adherent cells and then collected and counted manually in a Bürker chamber.

### Preparations of *T. gondii*-infected DCs for inoculation in mice

Preparations of cells for inoculations in mice were performed as previously described (15, 16). Briefly, DCs were pre-labeled with CMTMR (Invitrogen, Cat# C2927) and challenged with freshly egressed *T. gondii* tachyzoites for 4 h with MOI 2 to obtain an infection frequency of ~50%. Total numbers of cfu injected into animals were confirmed by plaquing assays. Host cell number and viability were assessed by hemocytometry.

### ip pre-infections of mice, ip treatments, plasma, and peritoneal lavage

For pre-ip infections, 6- to 8-week-old mice were inoculated ip with freshly egressed *T. gondii* tachyzoites (ME49-RFP). For rCCR5 pretreatments, mice were inoculated ip with rCCL5 (1 µg/mouse) 24 h prior to the experiment. For plasma sampling, blood was drawn into heparinized tubes by cardiac puncture and plasma fraction collected after sedimentation. Peritoneal fluid was collected by peritoneal lavage with 5 mL PBS.

### ICA inoculations and treatments

Mice (6 to 8 weeks old) were inoculated in the ICA and monitored as described (17). The medium containing cell/parasite suspensions (100 µL) was slowly injected over 5 min into the ICA. When indicated, mvc (10 µM) was added to DCs 1 h prior to injection. rCCL5 (1 µg/mouse) was injected ip 24 h before the ICA inoculation.

### Quantification of DCs and *T. gondii* foci in cortical brain sections

Brains were washed in PBS and fixed in 4% paraformaldehyde (PFA) for 48 h at 4°C. Then, PFA was replaced by PBS 10% sucrose (Sigma-Aldrich) for 2 d at 4°C. Fixed brains were frozen in liquid nitrogen, and 50-μm-thick cryosections were collected on glass slides. To quantify the number of infected DC/*T. gondii* foci, the prefrontal cortex area of the brain was imaged using epifluorescence microscopy (Leica DMi8). Data were expressed as the number of infected DCs per cm^2^ of tissue. Images were manually quantified with Fiji/ImageJ software. Data tables were exported and plotted using GraphPad Prism v.10 software.

### qPCR

Cells were cultured in DMEM or challenged with freshly egressed *T. gondii* tachyzoites of the indicated strains and lysed in Lysis buffer (Jena Bioscience). Total RNA was extracted according to the manufacturer’s protocol using the Total RNA Purification kit (Jena Bioscience) and reverse-transcribed with Maxima H Minus Reverse Transcriptase (Thermo Fisher). Real-time qPCR was performed with SYBR green PCR master mix (KAPA biosystems) or HotStart 2× SYBR Green qPCR Master Mix (APExBio Technology), specific forward and reverse primers at target-dependent concentrations (100–200 nM), and cDNA (10–15 ng) in a QuantStudio 5 System (Thermo Fisher) with ROX as a passive reference. qPCR results were analyzed using the ΔCt method relative to importin-8 and TATA-binding protein as housekeeping genes and displayed as fold change to unchallenged (set to 1). Primer sequences are listed in Table S1.

For brain micro-vessels, total RNA was extracted using the RNeasy mini kit (Qiagen, Cat# 74104) according to the manufacturer’s protocol. The RNA was quantified by spectrophotometry (NanoDrop 1000). cDNA was synthesized with Maxima First-Strand cDNA synthesis kit (ThermoFisher, Cat# EP0753). Real-time PCR was performed using 10–100 ng cDNA, 200 nM forward and reverse primers, and SYBR green PCR master kit (Kapa Biosystem). Primers are listed in Table S1. GAPDH was used as the housekeeping gene to generate ∆Ct values in order to calculate the relative expression (2^−ΔΔCT^).

### Statistical analyses

Statistical analyses were performed with Prism software (GraphPad v. 9). Hypothesis tests used are indicated in the figure legends and were chosen based on the experimental design, the hypothesis to be tested, data distribution, and which statistics were to be presented. In all tests, statistical significance is defined as *P* < 0.05.

## Data Availability

Requests for further information, resources, and reagents should be directed to and will be fulfilled by the corresponding author.

## SUPPLEMENTAL MATERIAL

Table S1 mbio.03444-25-s0001.pdf Key resources used in this study.

ASM does not own the copyrights to Supplemental Material that may be linked to, or accessed through, an article. The authors have granted ASM a non-exclusive, world-wide license to publish the Supplemental Material files. Please contact the corresponding author directly for reuse.

## References

[1] Kaplan L, Chow BW, Gu C. 2020. Neuronal regulation of the blood-brain barrier and neurovascular coupling. Nat Rev Neurosci 21:416–432. doi:10.1038/s41583-020-0322-2 32636528 PMC8934575

[2] Coureuil M, Lécuyer H, Bourdoulous S, Nassif X. 2017. A journey into the brain: insight into how bacterial pathogens cross blood-brain barriers. Nat Rev Microbiol 15:149–159. doi:10.1038/nrmicro.2016.178 28090076

[3] Zeng Z, Lan T, Wei Y, Wei X. 2022. CCL5/CCR5 axis in human diseases and related treatments. Genes Dis 9:12–27. doi:10.1016/j.gendis.2021.08.004 34514075 PMC8423937

[4] Griffith JW, Sokol CL, Luster AD. 2014. Chemokines and chemokine receptors: positioning cells for host defense and immunity. Annu Rev Immunol 32:659–702. doi:10.1146/annurev-immunol-032713-120145 24655300

[5] Aldinucci D, Borghese C, Casagrande N. 2020. The CCL5/CCR5 axis in cancer progression. Cancers (Basel) 12:1765. doi:10.3390/cancers12071765 32630699 PMC7407580

[6] Subileau EA, Rezaie P, Davies HA, Colyer FM, Greenwood J, Male DK, Romero IA. 2009. Expression of chemokines and their receptors by human brain endothelium: implications for multiple sclerosis. J Neuropathol Exp Neurol 68:227–240. doi:10.1097/NEN.0b013e318197eca7 19225413

[7] Lanfranco MF, Mocchetti I, Burns MP, Villapol S. 2017. Glial- and neuronal-specific expression of CCL5 mRNA in the rat brain. Front Neuroanat 11:137. doi:10.3389/fnana.2017.00137 29375328 PMC5770405

[8] Yao H, Jiang SY, Jiao YY, Zhou ZY, Zhu Z, Wang C, Zhang KZ, Ma TF, Hu G, Du RH, Lu M. 2025. Astrocyte-derived CCL5-mediated CCR5^+^ neutrophil infiltration drives depression pathogenesis. Sci Adv 11:eadt6632. doi:10.1126/sciadv.adt6632 40397747 PMC12094238

[9] Festa BP, Siddiqi FH, Jimenez-Sanchez M, Won H, Rob M, Djajadikerta A, Stamatakou E, Rubinsztein DC. 2023. Microglial-to-neuronal CCR5 signaling regulates autophagy in neurodegeneration. Neuron 111:2021–2037. doi:10.1016/j.neuron.2023.04.006 37105172

[10] Montoya JG, Liesenfeld O. 2004. Toxoplasmosis. Lancet 363:1965–1976. doi:10.1016/S0140-6736(04)16412-X 15194258

[11] Dubey JP. 1996. *Toxoplasma gondii*. In Baron S (ed), Medical microbiology, 4th ed. Galveston, TX. 21413265

[12] Courret N, Darche S, Sonigo P, Milon G, Buzoni-Gâtel D, Tardieux I. 2006. CD11c- and CD11b-expressing mouse leukocytes transport single Toxoplasma gondii tachyzoites to the brain. Blood 107:309–316. doi:10.1182/blood-2005-02-0666 16051744 PMC1895351

[13] Lambert H, Hitziger N, Dellacasa I, Svensson M, Barragan A. 2006. Induction of dendritic cell migration upon Toxoplasma gondii infection potentiates parasite dissemination. Cell Microbiol 8:1611–1623. doi:10.1111/j.1462-5822.2006.00735.x 16984416

[14] Weidner JM, Barragan A. 2014. Tightly regulated migratory subversion of immune cells promotes the dissemination of Toxoplasma gondii. Int J Parasitol 44:85–90. doi:10.1016/j.ijpara.2013.09.006 24184911

[15] Fuks JM, Arrighi RBG, Weidner JM, Kumar Mendu S, Jin Z, Wallin RPA, Rethi B, Birnir B, Barragan A. 2012. GABAergic signaling is linked to a hypermigratory phenotype in dendritic cells infected by Toxoplasma gondii. PLoS Pathog 8:e1003051. doi:10.1371/journal.ppat.1003051 23236276 PMC3516538

[16] Ten Hoeve AL, Braun L, Rodriguez ME, Olivera GC, Bougdour A, Belmudes L, Couté Y, Saeij JPJ, Hakimi M-A, Barragan A. 2022. The Toxoplasma effector GRA28 promotes parasite dissemination by inducing dendritic cell-like migratory properties in infected macrophages. Cell Host Microbe 30:1570–1588. doi:10.1016/j.chom.2022.10.001 36309013 PMC9710525

[17] Rodriguez ME, Hassan A, Linaroudis N, Harryson-Oliveberg F, Ten Hoeve AL, Barragan A. 2025. ICAM-1/CD18-mediated sequestration of parasitized phagocytes in cortical capillaries promotes neuronal colonization by Toxoplasma gondii. Nat Commun 16:3529. doi:10.1038/s41467-025-58655-z 40229286 PMC11997185

[18] Konradt C, Ueno N, Christian DA, Delong JH, Pritchard GH, Herz J, Bzik DJ, Koshy AA, McGavern DB, Lodoen MB, Hunter CA. 2016. Endothelial cells are a replicative niche for entry of Toxoplasma gondii to the central nervous system. Nat Microbiol 1:16001. doi:10.1038/nmicrobiol.2016.1 27572166 PMC4966557

[19] Olivera GC, Ross EC, Peuckert C, Barragan A. 2021. Blood-brain barrier-restricted translocation of Toxoplasma gondii from cortical capillaries. eLife 10:e69182. doi:10.7554/eLife.69182 34877929 PMC8700292

[20] Ross EC, Olivera GC, Barragan A. 2019. Dysregulation of focal adhesion kinase upon Toxoplasma gondii infection facilitates parasite translocation across polarised primary brain endothelial cell monolayers. Cell Microbiol 21:e13048. doi:10.1111/cmi.13048 31099453

[21] Afanaseva E, Barragan A. 2025. TIMP1 secretion induced by Toxoplasma effector GRA24 via p38 MAPK signaling promotes non-disruptive parasite translocation across polarized brain endothelial monolayers. mSphere 10:e00102-25. doi:10.1128/msphere.00102-25 40265926 PMC12108053

[22] Hakimi MA, Olias P, Sibley LD. 2017. Toxoplasma effectors targeting host signaling and transcription. Clin Microbiol Rev 30:615–645. doi:10.1128/CMR.00005-17 28404792 PMC5475222

[23] Franco M, Panas MW, Marino ND, Lee M-C, Buchholz KR, Kelly FD, Bednarski JJ, Sleckman BP, Pourmand N, Boothroyd JC. 2016. A novel secreted protein, MYR1, is central to Toxoplasma's manipulation of host cells. mBio 7:e02231-15. doi:10.1128/mBio.02231-15 26838724 PMC4742717

[24] Cygan AM, Theisen TC, Mendoza AG, Marino ND, Panas MW, Boothroyd JC. 2020. Coimmunoprecipitation with MYR1 identifies three additional proteins within the Toxoplasma gondii parasitophorous vacuole required for translocation of dense granule effectors into host cells. mSphere 5:e00858-19. doi:10.1128/mSphere.00858-19 PMC7031616 32075880

[25] Braun L, Brenier-Pinchart M-P, Hammoudi P-M, Cannella D, Kieffer-Jaquinod S, Vollaire J, Josserand V, Touquet B, Couté Y, Tardieux I, Bougdour A, Hakimi M-A. 2019. The Toxoplasma effector TEEGR promotes parasite persistence by modulating NF-κB signalling via EZH2. Nat Microbiol 4:1208–1220. doi:10.1038/s41564-019-0431-8 31036909 PMC6591128

[26] Rosowski EE, Lu D, Julien L, Rodda L, Gaiser RA, Jensen KDC, Saeij JPJ. 2011. Strain-specific activation of the NF-κB pathway by GRA15, a novel Toxoplasma gondii dense granule protein. J Exp Med 208:195–212. doi:10.1084/jem.20100717 21199955 PMC3023140

[27] Ten Hoeve AL, Rodriguez ME, Säflund M, Michel V, Magimel L, Ripoll A, Yu T, Hakimi M-A, Saeij JPJ, Ozata DM, Barragan A. 2024. Hypermigration of macrophages through the concerted action of GRA effectors on NF-κB/p38 signaling and host chromatin accessibility potentiates Toxoplasma dissemination. mBio 15:e02140-24. doi:10.1128/mbio.02140-24 39207098 PMC11481493

[28] Wickremasinghe MI, Thomas LH, O’Kane CM, Uddin J, Friedland JS. 2004. Transcriptional mechanisms regulating alveolar epithelial cell-specific CCL5 secretion in pulmonary tuberculosis. J Biol Chem 279:27199–27210. doi:10.1074/jbc.M403107200 15117956

[29] Bhandage AK, Olivera GC, Kanatani S, Thompson E, Loré K, Varas-Godoy M, Barragan A. 2020. A motogenic GABAergic system of mononuclear phagocytes facilitates dissemination of coccidian parasites. eLife 9:e60528. doi:10.7554/eLife.60528 33179597 PMC7685707

[30] Dorr P, Westby M, Dobbs S, Griffin P, Irvine B, Macartney M, Mori J, Rickett G, Smith-Burchnell C, Napier C, Webster R, Armour D, Price D, Stammen B, Wood A, Perros M. 2005. Maraviroc (UK-427,857), a potent, orally bioavailable, and selective small-molecule inhibitor of chemokine receptor CCR5 with broad-spectrum anti-human immunodeficiency virus type 1 activity. Antimicrob Agents Chemother 49:4721–4732. doi:10.1128/AAC.49.11.4721-4732.2005 16251317 PMC1280117

[31] Woollard SM, Kanmogne GD. 2015. Maraviroc: a review of its use in HIV infection and beyond. Drug Des Devel Ther 9:5447–5468. doi:10.2147/DDDT.S90580 PMC4598208 26491256

[32] Ross EC, Hoeve ALT, Saeij JPJ, Barragan A. 2022. Toxoplasma effector-induced ICAM-1 expression by infected dendritic cells potentiates transmigration across polarised endothelium. Front Immunol 13:950914. doi:10.3389/fimmu.2022.950914 35990682 PMC9381734

[33] Ólafsson EB, Barragan A. 2020. The unicellular eukaryotic parasite Toxoplasma gondii hijacks the migration machinery of mononuclear phagocytes to promote its dissemination. Biol Cell 112:239–250. doi:10.1111/boc.202000005 32359185

[34] Ross EC, Olivera GC, Barragan A. 2022. Early passage of Toxoplasma gondii across the blood-brain barrier. Trends Parasitol 38:450–461. doi:10.1016/j.pt.2022.02.003 35227615

[35] Costa RM, Cerqueira DM, Bruder-Nascimento A, Alves JV, Awata WMC, Singh S, Kufner A, Prado DS, Johny E, Cifuentes-Pagano E, Hawse WF, Dutta P, Pagano PJ, Ho J, Bruder-Nascimento T. 2024. Role of the CCL5 and its receptor, CCR5, in the genesis of aldosterone-induced hypertension, vascular dysfunction, and end-organ damage. Hypertension 81:776–786. doi:10.1161/HYPERTENSIONAHA.123.21888 38240165 PMC10954408

[36] Mukhopadhyay D, Arranz-Solís D, Saeij JPJ. 2020. Toxoplasma GRA15 and GRA24 are important activators of the host innate immune response in the absence of TLR11. PLoS Pathog 16:e1008586. doi:10.1371/journal.ppat.1008586 32453782 PMC7274473

[37] Ross EC, Ten Hoeve AL, Barragan A. 2021. Integrin-dependent migratory switches regulate the translocation of Toxoplasma-infected dendritic cells across brain endothelial monolayers. Cell Mol Life Sci 78:5197–5212. doi:10.1007/s00018-021-03858-y 34023934 PMC8254729

[38] Dyer DP, Medina-Ruiz L, Bartolini R, Schuette F, Hughes CE, Pallas K, Vidler F, Macleod MKL, Kelly CJ, Lee KM, Hansell CAH, Graham GJ. 2019. Chemokine receptor redundancy and specificity are context dependent. Immunity 50:378–389. doi:10.1016/j.immuni.2019.01.009 30784579 PMC6382461

[39] Yanaba K, Mukaida N, Matsushima K, Murphy PM, Takehara K, Sato S. 2004. Role of C-C chemokine receptors 1 and 5 and CCL3/macrophage inflammatory protein-1alpha in the cutaneous Arthus reaction: possible attenuation of their inhibitory effects by compensatory chemokine production. Eur J Immunol 34:3553–3561. doi:10.1002/eji.200425426 15517609

[40] Weidner JM, Kanatani S, Hernández-Castañeda MA, Fuks JM, Rethi B, Wallin RPA, Barragan A. 2013. Rapid cytoskeleton remodelling in dendritic cells following invasion by Toxoplasma gondii coincides with the onset of a hypermigratory phenotype. Cell Microbiol 15:1735–1752. doi:10.1111/cmi.12145 23534541

[41] Aliberti J, Valenzuela JG, Carruthers VB, Hieny S, Andersen J, Charest H, Reis e Sousa C, Fairlamb A, Ribeiro JM, Sher A. 2003. Molecular mimicry of a CCR5 binding-domain in the microbial activation of dendritic cells. Nat Immunol 4:485–490. doi:10.1038/ni915 12665855

[42] Ibrahim HM, Nishimura M, Tanaka S, Awadin W, Furuoka H, Xuan X, Nishikawa Y. 2014. Overproduction of Toxoplasma gondii cyclophilin-18 regulates host cell migration and enhances parasite dissemination in a CCR5-independent manner. BMC Microbiol 14:76. doi:10.1186/1471-2180-14-76 24661782 PMC3987834

[43] Marchetti L, Engelhardt B. 2020. Immune cell trafficking across the blood-brain barrier in the absence and presence of neuroinflammation. Vasc Biol 2:H1–H18. doi:10.1530/VB-19-0033 32923970 PMC7439848

[44] Kivisäkk P, Mahad DJ, Callahan MK, Trebst C, Tucky B, Wei T, Wu L, Baekkevold ES, Lassmann H, Staugaitis SM, Campbell JJ, Ransohoff RM. 2003. Human cerebrospinal fluid central memory CD4^+^ T cells: evidence for trafficking through choroid plexus and meninges via P-selectin. Proc Natl Acad Sci USA 100:8389–8394. doi:10.1073/pnas.1433000100 12829791 PMC166239

[45] Mordue DG, Monroy F, La Regina M, Dinarello CA, Sibley LD. 2001. Acute toxoplasmosis leads to lethal overproduction of Th1 cytokines. J Immunol 167:4574–4584. doi:10.4049/jimmunol.167.8.4574 11591786

[46] Wen X, Kudo T, Payne L, Wang X, Rodgers L, Suzuki Y. 2010. Predominant interferon-γ-mediated expression of CXCL9, CXCL10, and CCL5 proteins in the brain during chronic infection with Toxoplasma gondii in BALB/c mice resistant to development of toxoplasmic encephalitis. J Interferon Cytokine Res 30:653–660. doi:10.1089/jir.2009.0119 20626297 PMC2963637

[47] de Araújo TE, Coelho-Dos-Reis JG, Béla SR, Carneiro ACAV, Machado AS, Cardoso LM, Ribeiro ÁL, Dias MHF, Queiroz Andrade GM, Vasconcelos-Santos DV, Januário JN, Teixeira-Carvalho A, Vitor RWA, Ferro EAV, Martins-Filho OA, UFMG Congenital Toxoplasmosis Brazilian Group – UFMG-CTBG. 2017. Early serum biomarker networks in infants with distinct retinochoroidal lesion status of congenital toxoplasmosis. Cytokine 95:102–112. doi:10.1016/j.cyto.2017.02.018 28254558

[48] Denis J, Gommenginger C, Strechie T, Filisetti D, Beal L, Pfaff AW, Villard O. 2022. Dynamic immune profile in French toxoplasmosis patients. J Infect Dis 226:1834–1841. doi:10.1093/infdis/jiac305 35978487 PMC9650498

[49] Øynebråten I, Barois N, Bergeland T, Küchler AM, Bakke O, Haraldsen G. 2015. Oligomerized, filamentous surface presentation of RANTES/CCL5 on vascular endothelial cells. Sci Rep 5:9261. doi:10.1038/srep09261 25791723 PMC4367157

[50] Vilela MC, Mansur DS, Lacerda-Queiroz N, Rodrigues DH, Lima GK, Arantes RME, Kroon EG, da Silva Campos MA, Teixeira MM, Teixeira AL. 2009. The chemokine CCL5 is essential for leukocyte recruitment in a model of severe Herpes simplex encephalitis. Ann N Y Acad Sci 1153:256–263. doi:10.1111/j.1749-6632.2008.03959.x 19236348

[51] dos Santos AC, Barsante MM, Arantes RME, Bernard CCA, Teixeira MM, Carvalho-Tavares J. 2005. CCL2 and CCL5 mediate leukocyte adhesion in experimental autoimmune encephalomyelitis—an intravital microscopy study. J Neuroimmunol 162:122–129. doi:10.1016/j.jneuroim.2005.01.020 15833367

[52] Tyner JW, Uchida O, Kajiwara N, Kim EY, Patel AC, O’Sullivan MP, Walter MJ, Schwendener RA, Cook DN, Danoff TM, Holtzman MJ. 2005. CCL5-CCR5 interaction provides antiapoptotic signals for macrophage survival during viral infection. Nat Med 11:1180–1187. doi:10.1038/nm1303 16208318 PMC6322907

[53] Alonzo III F, Kozhaya L, Rawlings SA, Reyes-Robles T, DuMont AL, Myszka DG, Landau NR, Unutmaz D, Torres VJ. 2013. CCR5 is a receptor for Staphylococcus aureus leukotoxin ED. Nature 493:51–55. doi:10.1038/nature11724 23235831 PMC3536884

[54] Khan IA, Thomas SY, Moretto MM, Lee FS, Islam SA, Combe C, Schwartzman JD, Luster AD. 2006. CCR5 is essential for NK cell trafficking and host survival following Toxoplasma gondii infection. PLoS Pathog 2:e49. doi:10.1371/journal.ppat.0020049 16789839 PMC1475660

[55] Aliberti J, Reis e Sousa C, Schito M, Hieny S, Wells T, Huffnagle GB, Sher A. 2000. CCR5 provides a signal for microbial induced production of IL-12 by CD8α^+^ dendritic cells. Nat Immunol 1:83–87. doi:10.1038/76957 10881180

[56] Aliberti J, Jankovic D, Sher A. 2004. Turning it on and off: regulation of dendritic cell function in Toxoplasma gondii infection. Immunol Rev 201:26–34. doi:10.1111/j.0105-2896.2004.00179.x 15361230

[57] Schlecker E, Stojanovic A, Eisen C, Quack C, Falk CS, Umansky V, Cerwenka A. 2012. Tumor-infiltrating monocytic myeloid-derived suppressor cells mediate CCR5-dependent recruitment of regulatory T cells favoring tumor growth. J Immunol 189:5602–5611. doi:10.4049/jimmunol.1201018 23152559

[58] Schneider CA, Figueroa Velez DX, Azevedo R, Hoover EM, Tran CJ, Lo C, Vadpey O, Gandhi SP, Lodoen MB. 2019. Imaging the dynamic recruitment of monocytes to the blood-brain barrier and specific brain regions during Toxoplasma gondii infection. Proc Natl Acad Sci USA 116:24796–24807. doi:10.1073/pnas.1915778116 31727842 PMC6900744

[59] Schneider CA, Figueroa Velez DX, Orchanian SB, Shallberg LA, Agalliu D, Hunter CA, Gandhi SP, Lodoen MB. 2022. Toxoplasma gondii dissemination in the brain is facilitated by infiltrating peripheral immune cells. mBio 13:e02838-22. doi:10.1128/mbio.02838-22 36445695 PMC9765297

[60] Orchanian SB, Zolog S, Tomasello J, Malik R, Shin JH, Koshy AA, Lodoen MB. 2025. Toxoplasma gondii parasites induce a localized myeloid cell immune response surrounding parasites in the brain during acute infection. mBio 16:e00810-25. doi:10.1128/mbio.00810-25 40492741 PMC12239593

