# Supplementary material for: Toxoplasma effector GRA15-driven CCL5 secretion enhances brain parasite load through microvascular sequestration of phagocytes
Source: mBio. 2026 Jan 13;17(2):e03444-25. doi: 10.1128/mbio.03444-25 (PMC12893009; doi:10.1128/mbio.03444-25)
Supplement: Table S1 — Key resources used in this study. [file mbio.03444-25-s0001.pdf]

Table S1. Key resources used in the study

REAGENT or RESOURCE	SOURCE	IDENTIFIER
**Chemicals and recombinant proteins**		
JSH-23 p65 inhibitor	MedChemExpress	Cat# HY-13982
TPCA-1 IKK2, STAT3 inhibitor	MedChemExpress	Cat# HY-10320
Maraviroc CCR5 inhibitor	MedChemExpress	Cat# HY-13004
Dextran Fluorescein 3000 MW anionic	Thermo Fisher	Cat# D3305
Dextran from *Leuconostoc spp.* 70000 MW	Sigma Aldrich	Cat# 31390
Recombinant Mouse CCL5 Protein CF	Peprotech	Cat# 250-07
Recombinant Mouse GM-CSF	Peprotech	Cat# 315-03
CellTracker Orange CMTMR Dye	Invitrogen	Cat# C2927
**Commercial assays**		
Total RNA purification kit	Jena Bioscience	Cat# PP-210L
Mouse RANTES instant ELISA Kit	Invitrogen	Cat# BMS6009INST
**Cell lines**		
Human foreskin fibroblasts HFF-1	American Type Culture Collection	Cat# SCRC-1041 RRID:CVCL_3285
bEnd.3	American Type Culture Collection	Cat# ATCC CRL-2299 RRID:CVCL_0170
**Parasite strains**		
T. gondii RH-LDM GFPS65T	(Kim et al., 2001; Barragan and Sibley, 2002)	N/A
T. gondii RH1-1 cLuc GFP$^+$	(Boyle et al., 2007)	N/A
T. gondii RH Δ*myr1* cLuc GFP$^+$	(Wang et al., 2019)	N/A
T. gondii Pru Δ*hpt* GFP$^+$ (PRU A7)	(Kim et al., 2007)	N/A
T. gondii Pru Δ*hpt* GFP$^+$ Δ*gra15*	(Mukhopadhyay et al., 2020)	N/A
T. gondii Pru GFP$^+$ Δ*gra15+gra15*	(Rosowski et al., 2011)	N/A
T. gondii Pru Δ*ku80*	(Braun et al., 2019)	N/A
T. gondii Pru Δ*ku80* Δ*teegr*	(Braun et al., 2019)	N/A
T. gondii Pru Δ*ku80* Δ*myr1*	(Braun et al., 2019)	N/A
**Software and algorithms**		
ImageJ	(Schneider et al., 2012)	https://imagej.nih.gov/ij/
GraphPad Prism 9.0	GraphPad Software	http://www.graphpad.com
**qPCR primers**		
Target	Sequence 5'-3'	
mouse *Ccl5* fw	ATATGGCTCGGACACCACTC	
mouse *Ccl5* rv	TTCGAGTGACAAACACGACTG	
mouse *Ipo8* fw	CTATGCTCTCGTTCAGTATGC	
mouse *Ipo8* rv	GTCCGAAAGATCTCCATCCA	
mouse *Tbp* fw	GGGGAGCTGTGATGTGAAGT	
mouse *Tbp* rv	CCAGGAAATAATTCTGGCTCA	
mouse *Gapdh* fw	TGACCTCAACTACATGGTCTACA	
mouse *Gapdh* rv	CTTCCCATTCTCGGCCTTG	

mouse *Icam-1* fw	CAATTTCTCATGCCGCACAG	
mouse *Icam-1* rv	CTGGAAGATCGAAAGTCCGG	
mouse *Vcam-1* fw	GTGACTCCATGGCCCTCACTT	
mouse *Vcam-1* rv	CGTCCTCACCTTCGCGTTTA	
mouse *Elam* fw	CCCTGCCCACGGTATCAG	
mouse *Elam* rv	ACGTGCATGTCGTGTTCCA	
T. gongii B1 fw	GCATTGCCCGTCCAAACT	
T. gongii B1 rv	AGACTGTACGGAATGGAGACGAA	

Barragan, A., and L.D. Sibley. 2002. Transepithelial migration of Toxoplasma gondii is linked to parasite motility and virulence. *J Exp Med* 195:1625-1633.

Boyle, J.P., J.P. Saeij, and J.C. Boothroyd. 2007. Toxoplasma gondii: inconsistent dissemination patterns following oral infection in mice. *Exp Parasitol* 116:302-305.

Braun, L., M.P. Brenier-Pinchart, P.M. Hammoudi, D. Cannella, S. Kieffer-Jaquinod, J. Vollaire, V. Josserand, B. Touquet, Y. Couté, I. Tardieux, A. Bougdour, and M.A. Hakimi. 2019. The Toxoplasma effector TEEGR promotes parasite persistence by modulating NF-κB signalling via EZH2. *Nat Microbiol* 4:1208-1220.

Kim, K., M.S. Eaton, W. Schubert, S. Wu, and J. Tang. 2001. Optimized expression of green fluorescent protein in Toxoplasma gondii using thermostable green fluorescent protein mutants. *Mol Biochem Parasitol* 113:309-313.

Kim, S.K., A. Karasov, and J.C. Boothroyd. 2007. Bradyzoite-specific surface antigen SRS9 plays a role in maintaining Toxoplasma gondii persistence in the brain and in host control of parasite replication in the intestine. *Infection and immunity* 75:1626-1634.

Mukhopadhyay, D., D. Arranz-Solís, and J.P.J. Saeij. 2020. Toxoplasma GRA15 and GRA24 are important activators of the host innate immune response in the absence of TLR11. *PLoS pathogens* 16:e1008586.

Rosowski, E.E., D. Lu, L. Julien, L. Rodda, R.A. Gaiser, K.D. Jensen, and J.P. Saeij. 2011. Strain-specific activation of the NF-kappaB pathway by GRA15, a novel Toxoplasma gondii dense granule protein. *The Journal of experimental medicine* 208:195-212.

Schneider, C.A., W.S. Rasband, and K.W. Eliceiri. 2012. NIH Image to ImageJ: 25 years of image analysis. *Nature methods* 9:671-675.

Wang, Y., K.M. Cirelli, P.D.C. Barros, L.O. Sangare, V. Butty, M.A. Hassan, P. Pesavento, A. Mete, and J.P.J. Saeij. 2019. Three Toxoplasma gondii Dense Granule Proteins Are Required for Induction of Lewis Rat Macrophage Pyroptosis. *mBio* 10:
